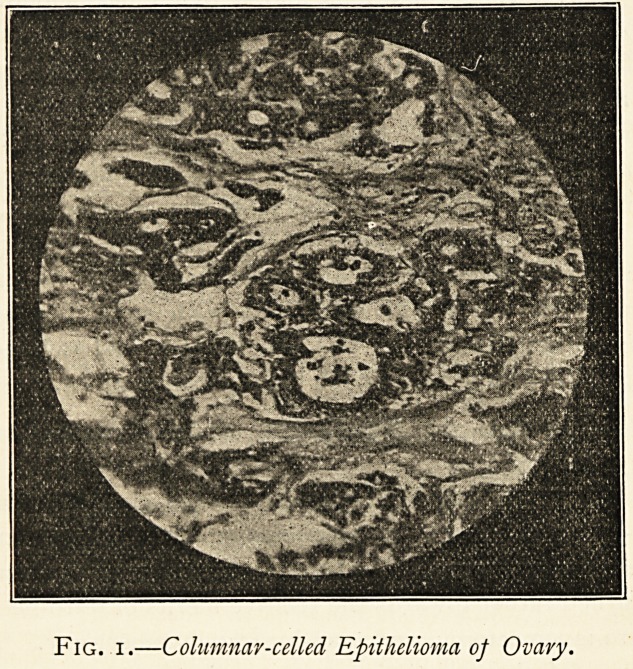# A Retrospect of a Third Series of Fifty Consecutive Intra-Abdominal Operations

**Published:** 1901-03

**Authors:** James Swain

**Affiliations:** Professor of Surgery at University College, Bristol; Assistant-Surgeon to the Bristol Royal Infirmary.


### A RETROSPECT OF A THIRD SERIES OF FIFTY CONSECUTIVE INTRA-ABDOMINAL OPERATIONS.

BY

James Swain, M.S., M.D. Lond., F.R.C.S. Eng.,

*Professor of Surgery at University College, Bristol; Assistant-Surgeon to the Bristol Royal Infirmary.*

I PREFER to present this series of cases in the same form as before, leaving those who have a mind for statistics to deal with them in such manner as pleases them. The table given below is simply arranged chronologically, without any attempt at other classification.

This series is continuous with the former two,[1] and the same limitation (as to the exclusion of operations upon the kidney, bladder, etc.) has been adopted as before. The most interesting features are perhaps to be found in the variety of some (*e.g.* cases 24, 30, etc.) conditions, and the complexity of others (*e.g.* cases 28, 37, etc.): at all events there was more than sufficient provision for such diagnostic acumen and manipulative dexterity as one might possess; and if the number of cases quoted is large, I trust that the notes will contain enough of value to render an apology unnecessary.

In discussing these cases it is convenient to group together those of a similar nature; but details are given only in those which present some important feature.

*A*.—**Operations for Diseases of the Ovaries and Fallopian Tubes.** —CASES 5, 10, 12, 14, 17, 18, 21, 27, 28, 30, 32, and 37.

Clinically, this group may be divided into—

**Adeno-cystoma.**—CASES 5, 10, 14, 18, 27 and 32.

**Intra-ligamentary Cyst.**—CASE 30.

**Salpingo-oöphoritis and Tubo-ovarian Abscess.**—CASES 12, 28 and 37.

**Columnar Epithelioma of Ovary.**—CASE 21.

**Oöphoritic Cyst and Dysmenorrhœa.**—CASE 17.

Of the **adeno-cystomata,** the opposite ovary had undergone

[1] *Bristol M.-Chir. J.*, 1898, xvi. 211; 1900, xviii. 103.

cystic degeneration to such an extent as to necessitate removal in four cases.

Attacks of pain and vomiting, especially if accompanied by collapse, in association with ovarian cysts are frequently dependent upon **twisting of the pedicle.** Such symptoms are of serious import, for unless speedy relief is afforded by operation death from suppuration and peritonitis may follow. Strangulation from torsion is more likely to occur in tumours possessing a long pedicle: the twisting is probably caused by peristaltic movements of the intestines. An example is afforded in the following case of a small tumour with a long pedicle moving freely among the intestines:—

CASE 27.—**Ovarian Adeno-cystoma with Twisted Pedicle.**—The patient had had two attacks of violent pain in the right side of the abdomen, accompanied by vomiting. When first seen she was pale, rather collapsed, and constantly complaining of pain in the right side and shooting down the legs. Temperature 99.2. A tumour about the size of the closed fist was found below and to the right of the umbilicus, and it was so freely movable that it could be placed in almost any part of the abdomen below a line joining the cartilages of the ninth rib. It could also be pressed down into the pelvis to the right side of the uterus, which was normal in size. Operation showed the tumour to be a right-sided ovarian adeno-cystoma with a long pedicle which was twisted for one complete turn. Some of the cysts contained clear yellow fluid, but here and there the contents were turbid. The cyst-wall contained dark, scattered patches from hemorrhage. The left ovary was also cystic. Both ovaries were removed, and the patient made an uninterrupted recovery.

A few interesting features of some of the other cases of **adeno-cystoma** may be briefly referred to:—

CASE 14 was associated with a milky fluid in each breast, though there were no other signs of pregnancy.

CASE 5 actually believed herself to be pregnant, as the tumour commenced soon after marriage. She subsequently became pregnant, and a child was born about thirteen months after marriage; and after this the tumour became larger and was tapped. This tapping was doubtless responsible for the adhesions to the anterior parietes which I found on performing ovariotomy five weeks after the birth of the child.

CASE 32 was an enormous tumour causing dulness over the whole abdomen, except where the intestines were pushed up in the epigastrium and annulled the liver dulness. The patient was almost pulseless, and it was only by operating rapidly that a successful result ensued. Adhesions—probably due to a previous tapping—were present over the entire front of the cyst wall. The cyst contained 27½ pints of an opaque yellowish-green alkaline fluid, sp. gr. 1022, containing 3.2 per cent. of albumin, .6 per cent. of urea, and some para-globulin and serum albumin.

### FIFTY CONSECUTIVE INTRA-ABDOMINAL OPERATIONS.

| No. | Sex and Age. | Medical Attendant. | Disease. | Treatment. | Result. |
|---|---|---|---|---|---|
| 1 | M. 68 ... | ... ... ... ... | Carcinoma of hepatic flexure of colon | Colostomy ... ... ... ... | Died. |
| 2 | M. 11 ... | Dr. H. Skelton ... | Appendicitis. Perforation of gut | Cœliotomy—drainage ... ... | Died. |
| 3 | F. 32 ... | Dr. J. Ambrose ... | Sinus following appendicitis ... | Cœliotomy—tamponnage ... ... | Recovered. |
| 4 | M. 61 ... | Mr. J. D. Staple ... | Volvulus. Intestinal obstruction... | Liberation of adhesions ... ... | Recovered. |
| 5 | F. 21 ... | Mr. E. F. Clowes ... | Ovarian adeno-cystoma (left)—cystic ovary (right) | Ovariotomy (left)—salpingo-oöphorectomy (right) ... ... ... | Recovered. |
| 6 | F. 54 ... | ... ... ... ... | Cirrhosis of liver ... ... ... | Hepatopexy ("Morison's operation") | Died. |
| 7 | M. 55 ... | Mr. T. M. Carter ... | Malignant stricture of rectum ... | Colostomy ... ... ... ... | Recovered. |
| 8 | F. 15 ... | Dr. E. B. Garland ... | Appendicitis .. ... ... ... | Abdomino-vaginal drainage ... | Recovered. |
| 9 | F. 22 ... | Dr. Macrae ... ... | Perforating gastric ulcer ... .. | Gastrorraphy ... ... ... | Died. |
| 10 | F. 35 ... | Mr. J. H. Parry ... | Ovarian cyst (universally adherent) | Exploratory cœliotomy ... ... | Recovered. |
| 11 | F. 33 ... | Dr. E. L. Lees... ... | Appendicitis ... ... ... ... | Appendicectomy ... ... ... | Recovered. |
| 12 | F. 35 ... | Mr. C. J. Perrott ... | ? Tubo-ovarian abscess (left). Intestinal perforation... ... ... | Cœliotomy—drainage ... ... | Recovered. |
| 13 | F. 22 ... | Dr. J. C. Maclean ... | Intestinal colic ... ... ... | Exploratory cœliotomy ... ... | Recovered. |
| 14 | F. 40 ... | Mr. E. H. Openshaw ... | Ovarian adeno-cystoma (right)—cystic ovary (left) ... ... | Ovariotomy (right) — salpingo-oöphorectomy (left) | Recovered. |
| 15 | F. 24 ... | Dr. R. Roxburgh ... | Appendicitis ... ... ... ... | Appendicectomy ... ... ... | Recovered. |
| 16 | F. 41 ... | Dr. J. C. Maclean ... | Tubercular peritonitis ... ... | Cœliotomy ... .. ... ... | Recovered. |
| 17 | F. 26 ... | Dr. J. C. Maclean ... | Dysmenorrhœa—cystic ovary (right) | Salpingo-oöphorectomy (double) ... | Recovered. |
| 18 | F. 49 ... | Dr. S. H. McQuade ... | Ovarian adeno-cystoma (left) ... | Ovariotomy ... ... ... ... | Recovered. |
| 19 | F. 42 ... | Dr. A. B. Blair ... | Myo-fibroma of uterus ... ... | Retro-peritoneal hystero-myomectomy | Recovered. |
| 20 | F. 35 ... | Mr. C. J. Perrott ... | Intestinal fistula ... ... ... | Jejunorraphy... ... ... ... | Recovered. |
| 21 | F. 26 ... | Dr. E. F. Martin ... | Columnar epithelioma of ovary (right) ... ... ... ... | Ovariectomy... ... ... ... | Recovered. |
| 22 | M. 65 ... | Dr. R. Roxburgh ... | Cholelithiasis ... ... ... | Cholecystotomy ... ... ... | Recovered. |
| 23 | F. 15 ... | Dr. E. B. Garland ... | Appendicitis ... ... ... ... | Appendicectomy ... ... ... | Recovered. |
| 24 | F. 28 ... | Dr. H. S. Ballance .. | Polypoid fibroma of pylorus ... | Excision of growth—pylorectomy | Recovered. |
| 25 | M 18 ... | Mr. J. Dacre ... ... | Rupture of intestine ... ... | Jejunorraphy... ... ... ... | Died. |

| No. | Sex and Age. | Medical Attendant. | Disease. | Treatment. | Result. |
|---|---|---|---|---|---|
| 26 | M. 42 ... | Mr. B. P. B. Burroughs | Appendicitis ... ... ... ... | Appendicectomy ... ... ... | Recovered. |
| 27 | F. 25 .. | ... ... ... ... | Ovarian adeno-cystoma (double) ... | Salpingo-oöphorectomy (double) ... | Recovered. |
| 28 | F. 28 ... | Dr. S. Irwin ... ... | Tubo-ovarian abscess. Appendicitis | Appendicectomy. Salpingo-oöphorectomy (double). Abdomino-vaginal drainage | Recovered. |
| 29 | M. 50 ... | ... ... ... ... | Malignant stricture of rectum ... | Colostomy ... ... ... ... | Recovered. |
| 30 | F. 29 ... | Dr. C. P. Crouch ... | Multiple cysts of broad ligaments | Enucleation of cysts ... ... | Recovered. |
| 31 | F. 48 ... | Dr. J. M. Rattray ... | Myo-fibroma of uterus ... ... | Exploratory cœliotomy — "morcellement" (vaginal) .. ... | Recovered. |
| 32 | F. 41 ... | Mr. Bunbury ... ... | Ovarian adeno-cystoma (left) — cystic ovary (right) ... ... | Ovariotomy (left)—salpingo-oöphorectomy (right) | Recovered. |
| 33 | F. 44 ... | Mr. J. S. Griffiths ... | Tumour of pancreas ... ... | Exploratory cœliotomy ... ... | Recovered. |
| 34 | F. 33 ... | Mr. G. T. Myles ... | Malignant stricture of rectum ... | Colostomy ... ... ... ... | Died. |
| 35 | F. 47 ... | Mr. H. T. Rudge ... | Myo-fibroma of uterus. Malignant disease of peritoneum ... ... | Exploratory cœliotomy ... ... | Recovered. |
| 36 | F. 39 ... | Mr. R. L. Willcox ... | Malignant disease of peritoneum... | Exploratory cœliotomy ... ... | Recovered. |
| 37 | F. 30 ... | Mr. D. Bradley ... | Salpingo-oöphoritis. Appendicitis | Salpingo-oöphorectomy. Appendicectomy | Recovered. |
| 38 | F. 22 ... | Mr. H. T. Rudge ... | Appendicitis ... ... ... ... | Appendicectomy .. ... ... | Recovered. |
| 39 | F. 78 ... | Dr. G. Parker .. ... | Carcinoma of descending colon ... | Cœliotomy ... .. ... ... | Recovered. |
| 40 | M. 9 ... | Dr. C. Matthews ... | Appendicitis ... ... ... ... | Drainage ... ... ... ... | Recovered. |
| 41 | M. 14 ... | ... ... ... ... | Perforation of intestine by a pin ... | Removal of foreign body—drainage | Recovered. |
| 42 | M. 25 ... | ... ... ... ... | Appendicitis ... ... ... ... | Appendicectomy ... ... ... | Recovered. |
| 43 | F. 44 ... | Dr. S. Irwin ... ... | Myo-fibroma of uterus ... ... | Retro-peritoneal hystero-myomectomy | Died. |
| 44 | M. 38 ... | Dr. H. Cook ... ... | Appendicitis ... ... ... ... | Appendicectomy ... ... ... | Recovered. |
| 45 | F. 44 ... | Dr. J. C. Maclean ... | Cholelithiasis ... ... ... | Cholecystotomy ... ... ... | Recovered. |
| 46 | M. 12 ... | ... ... ... ... | Appendicitis ... ... ... ... | Appendicectomy ... ... ... | Recovered. |
| 47 | F. 32 ... | Dr. J. Wallace... ... | Appendicitis ... ... ... ... | Appendicectomy ... ... ... | Recovered. |
| 48 | M. 21 ... | Dr. J. Michell Clarke ... | Appendicitis ... ... ... ... | Appendicectomy ... ... ... | Recovered. |
| 49 | F. 37 ... | Dr. E. F. Martin ... | Myo-fibroma of uterus ... ... | Pan-hysterectomy ... ... ... | Died. |
| 50 | F. 70 ... | Dr. R. Shingleton Smith | Carcinoma of gall-bladder and pylorus ... ... ... ... | Exploratory cœliotomy ... ... | Recovered. |

**Intra-ligamentary cysts**, other than those arising from the growth of ovarian tumours between the layers of the broad ligament, are little understood. Until I operated upon the case mentioned below, the existence of multiple intra-ligamentary cysts was unknown to me. Mr. Alban Doran, to whom I wrote on the subject, kindly informs me that he believes "that these endothelium-bearing cysts . . . are lymphatic" in origin. "The common broad ligament cyst bears epithelium."

CASE 30.—**Multiple Intra-ligamentary Cysts—Enucleation.**—Seven years before I saw her the patient was supposed to have a "hæmatocele"; and about five years after this Mr. Greig Smith performed a median cœliotomy, but closed the wound because of the presence of "tubercles." Ten months ago she had an attack of right abdominal pain, vomiting, and temperature, and was in bed for five weeks. A similar attack occurred about seven weeks ago, and the patient was still confined to bed with a temperature ranging from 98° to 103°. There had been a discharge of pus from the rectum for about four years, and amenorrhœa had existed for eighteen months. The patient looked ill and anæmic. There was tenderness in the right iliac fossa and a globular fluctuating swelling was felt in the mid-line reaching to within two inches of the umbilicus. *Per vaginam* and *per rectum* all the pelvic organs were fixed by a dense infiltration: there was slight bulging in the right lateral vaginal fornix. An incision was made at the site of the former operation, between the umbilicus and pubes. The omentum was firmly adherent to the parietes and to the pelvic wall; but on detaching this, a cyst appeared in the mid-line covered by an "extra-capsule" of broad ligament. This capsule was peeled off the true cyst, which was then tapped, and about six ounces of a turbid, greenish-yellow fluid (sp. gr. 1030, alkaline, cholesterin .2 per cent., urea .9 per cent., albumin 6.6 per cent., traces of phosphates) escaped with cholesterin crystals floating through it. A second cyst then appeared, attached to but independent of the first, and about the size of a Tangerine orange; this was similarly tapped and contained a clearer fluid. Two other smaller cysts appeared in like manner, but these burst in the process of enucleation. The collapsed cyst-walls were then completely enucleated from the capsule of broad ligament, and in so doing two very small cysts were broken into at the deeper part. The enucleation left a cavity the size of a cricket ball below and behind which the right Fallopian tube and ovary could apparently be felt embedded in dense inflammatory tissue. A cyst as large as a pigeon's egg, and containing clear fluid, was enucleated whole from the left broad ligament in a manner similar to the removal of the cysts from the right broad ligament. The floor of the pelvis was densely infiltrated with inflammatory deposit, and the top and front of the uterus were covered with adherent coils of intestine, mainly sigmoid flexure. Part of the right broad ligament capsule was cut away, and the rest sewn together with a continuous stitch so as to leave no raw surface to form adhesions. Abdomen closed without drainage. The adhesions throughout were very dense, and the operation accordingly difficult, though much facilitated by the adoption of the Trendelenburg posture. A good recovery followed, though convalescence was retarded by the necessity of draining an abscess associated with the old rectal trouble.

**Malignant ovarian tumours** are doubtless more common than one would suppose by microscopical appearances only; and Kelly[1] considers it desirable to regard all ovarian tumours as malignant until removed and proved otherwise. Rapid growth and the presence of a blood-stained peritoneal fluid, as in the case below, are always suggestive of malignancy.

CASE 21.—**Columnar Epithelioma of Ovary—Ovariectomy.**—The patient had been in ill health with slight abdominal pain for five months. Seven weeks before I saw her a tumour was discovered in the abdomen reaching as high as the umbilicus, and had rapidly increased. There was no loss of flesh. The temperature had varied between 99° and 100°. Menstruation was regular. The patient presented a healthy appearance. The abdomen was occupied by a large tumour producing a greater prominence on the right side than the left. The tumour had

FIG. 1.—*Columnar-celled Epithelioma of Ovary.*

the usual characteristics of an ovarian neoplasm, was fairly smooth, movable, semi-fluctuating, and reached a point half-way between the umbilicus and the ensiform cartilage. There were signs of fluid in the right chest. A six-inch incision was made in the mid-line reaching from two inches above the umbilicus nearly to the pubes. On opening the peritoneum a good deal of blood-stained fluid escaped. A soft, whitish-grey tumour presented and was delivered through the incision, and removed after tying the pedicle. The abdomen was closed without

[1] Kelly's *Operative Gynecology*, vol. ii., 1898, p. 248.

drainage, and the patient did well as far as the operation was concerned, but succumbed later to a secondary deposit which produced intestinal obstruction. The tumour removed weighed nearly eight pounds. On cutting into it, it was found to be vascular, reddish-grey in colour, and of the consistence of brain-matter. In one part was a degenerated yellowish mass half as large as a cocoa-nut, and containing about two ounces of yellowish fluid. A portion of the tumour was sent to the Clinical Research Association, which reported that "the tumour is a very soft and rapidly growing carcinoma of the ovary of the tubular columnar-celled class." This is well seen in the photomicrograph (Fig. 1) kindly prepared for me by Mr. James Taylor.

**Salpingo-oöphorectomy for small cystic ovaries** associated with dysmenorrhœa is not an operation which can be considered justifiable except in occasional cases, and for epilepsy it may be discarded as useless. Dysmenorrhœa is a symptom dependent upon many various conditions, and it by no means follows that the patient—who is frequently of the neurotic type—is really benefited by the removal of the ovaries; for though menstruation may cease, other nervous phenomena may be substituted for the dysmenorrhœa. It is not surprising, therefore, that the results of these operations have not been satisfactory. An incomplete but highly acceptable relief was afforded in Case 17, upon whom I performed salpingo-oöphorectomy, after watching the case for many months and satisfying myself that the enlarged cystic ovary was associated with such intense pain during menstruation that the patient, who had suffered from epilepsy since puberty, dreaded the coming-on of the "periods."

*B*.—**Operations for Myo-fibroma of the Uterus.**—Cases 19, 31, 35, 43, 49.

There is no longer any need to urge the advantages of intra-peritoneal over extra-peritoneal treatment of the stump in hystero-myomectomy. The tendency of more recent discussions has turned rather towards the relative merits of retro-peritoneal hystero-myomectomy and pan-hystero-myomectomy. In the presence of the association with malignant disease, nothing short of the removal of the whole uterus and tumour would be desirable; but in ordinary uncomplicated cases of hystero-myomectomy there is no *primâ facie* reason for the removal of any more of the uterus and tumour than is included in the supra-vaginal operation of retro-peritoneal hystero-myomectomy.

But though we may regard the last-mentioned operation as the method of election, it would be foolish to insist on a slavish adherence to it under all circumstances. In Case 49, for example, the tumour, which weighed five pounds, grew entirely from the right side of the uterus—the uterus being represented by a canal about one and a half inches in diameter on the left side of the tumour—and the lower end was so large and globular that a retro-peritoneal hystero-myomectomy would have left a very large stump to cover in, and I, therefore, deemed it best to perform pan-hystero-myomectomy.

There is no great difficulty in the "complete" operation. The preliminary steps are the same as those undertaken for retro-peritoneal hystero-myomectomy, which I described in a former paper.[1] After the uterine and ovarian vessels have been tied, a circular incision is made in the top of the vagina around the tumour, which is then removed entire. The vagina is closed, and the flaps of peritoneum which have been peeled off the back and front of the tumour are sewn together over it. The adoption of the Trendelenburg posture greatly facilitates the necessary manipulations.

The presence of a large myomatous uterus generally prevents conception or produces abortion. Operative interference for myo-fibromata associated with pregnancy should only be undertaken if it seems probable that the tumour will prevent the delivery of the child at full term. In the following case, which is interesting because of the difficulty in diagnosis, the existence of a calcareous fibroid, low down at the side of the uterus, would probably have impeded labour, though a normal pregnancy was not diagnosed till after operation.

CASE 19.—The patient had had seven children—two being stillborn at full time. Nine months before I saw her a tumour was noticed on the right side of the abdomen. At that time she was seven months pregnant, and the child was born two months later. After the birth of the child the tumour steadily increased in size. Menstruation recurred three months after parturition, and was profuse. Amenorrhœa had existed for four months; but the patient did not think she was pregnant. There was a more or less globular tumour in the mid-line of the abdomen reaching nearly to the umbilicus. On the right of this, in the pelvis, but reaching nearly to the right anterior superior spine, was a very hard globular mass (calcareous fibroid) larger than the closed fist.

[1] *Bristol M.-Chir. J.*, 1900, xviii. 110.

The os uteri was patulous, but not soft; the uterus appeared to be enlarged, and moved with the abdominal tumour. There was milk in the breasts. A skiagram gave negative results. A diagnosis was made of uterine myo-fibroma, possibly associated with an extra-uterine fœtation. A retro-peritoneal hystero-myomectomy was performed in the usual way. The myo-fibroma was calcareous throughout, and the uterus contained a fœtus in normal position. The patient made a good recovery.

*C.*—**Operation for Tubercular Peritonitis.**—Case 16.

The beneficial effect of operation on cases of chronic tubercular peritonitis is insufficiently appreciated. More females are operated upon than males, owing to a repetition of the initial error of Sir Spencer Wells in mistaking such a case for one of ovarian cyst; and such mistakes almost always turn out satisfactorily for the patient. It is unfortunate, from this point of view, that there is no disease in males similar to an ovarian cyst, for the good results of cœliotomy for tubercular peritonitis might then occasionally be seen in men. As matters stand at present, a large number of females are cured by operation; whereas the males are more often seen in the *post-mortem* room, without having had the chance of relief which the operation affords.[1]

The **diagnosis** between ovarian cyst and tubercular peritonitis is effected by observing the irregular relation of the areas of resonance and dulness (*e.g.* resonance over the pubes is common in tubercular peritonitis, and rare in ovarian cyst), the presence of fever, and the rapid accumulation of the fluid. All these were present in the case reported below.

Only the chronic forms are suitable for operation; and, speaking generally, the greater the amount of fluid (as in the ascitic variety) the greater the probability of cure. The operation is of the simplest possible character in many cases. The peritoneum is opened by incision, the fluid removed, and the wound then closed. No drainage is necessary, and flushing with antiseptics is superfluous. Treatment by aspiration is dangerous, because of the frequency of the existence of adherent intestines, and it is not so successful as cœliotomy.

It is scarcely needful to state that no operation should be undertaken if the tubercular peritonitis forms only a part of a

[1] Watson Cheyne's *Treatment of Tuberculous Diseases*, 1900, p. 54.

general tuberculosis. Many theories have been advanced to explain the successful results of cœliotomy for this condition, but none of them is entirely satisfactory.

Case 16.—**Tubercular Peritonitis—Cœliotomy.**—A period of ill health was followed, about six weeks before the patient came under observation, by pain in the left side of the abdomen, and a gradually increasing abdominal fulness especially marked for the past fortnight. There was much emaciation, the patient weighing only a little over five stone. The lower abdomen was tense and prominent, and gave an indistinct sensation of fluctuation and thrill as high as two inches above the umbilicus. There was resonance above the pubes and in various parts over the front of the swelling. Signs of free fluid were shown by the alteration of dulness in the flanks from alteration of posture. The temperature was 101°. On opening the abdomen between the umbilicus and pubes, a large quantity of yellowish fluid (sp. gr. 1020, neutral) escaped. The intestines were much congested, and the parietal peritoneum was of a deep red colour. The surface of the intestine, uterus, etc., and the parietal peritoneum was very rough from innumerable deposits of miliary tubercle as far as the finger could reach. The intestines were matted together above and to the left, forming a sort of cavity by which the fluid was largely contained in the right iliac fossa, though the general peritoneal cavity was not entirely shut off. The abdominal cavity was sponged out to remove the remainder of the fluid, and the wound closed without flushing or drainage. Recovery was rapid, and six months after operation the patient weighed nearly seven stone, and was "better in health than she had been for a long time."

### *D.*—**Hepatopexy for the Ascites of Cirrhosis.**—Case 6.

The bad results of paracentesis in cases of cirrhosis of the liver led Dr. Drummond and Mr. Rutherford Morison[1] to adopt the artificial production of adhesions with a view to relieving the portal circulation in these cases. Some time since[2] I epitomised the more recent views of Dr. Rolleston and Mr. Turner on this subject. It would appear to be important that operation should be undertaken earlier than heretofore, partly because patients with cirrhosis are prone to peritonitis after operation—and this was the cause of death in the case narrated below—and partly because the liver tissue is not likely to undergo a compensatory hyperplasia as a result of the improved blood-supply, unless operation is undertaken before marked degenerative changes have occurred. It must, however, be confessed that the success which has attended this operation

[1] *Brit. M. J.*, 1896, ii. 728.
[2] *Bristol M.-Chir. J.*, 1900, xviii. 39.

so far leaves a good deal to be desired. The procedure is explained in the following case:—

CASE 6.—**Morison's Operation for the Ascites of Cirrhosis.**—The patient had been tapped many times over a long period, and as much as thirty pints and twelve pints respectively had been drawn off at the last two tappings within a fortnight of the operation. A vertical incision three inches long was made in the right semi-lunar line, commencing just below the costal margin. About twenty pints of ascitic fluid escaped on opening the peritoneum. The abdominal cavity was then dried with sponges. The antero-superior surface of both lobes of the liver and the under surface of the diaphragm were then lightly scraped with a Volkmann's spoon. The omentum was looped up between the diaphragm and liver, and a catgut ligature was passed through the edge of the liver, the omentum and the cut parietal peritoneum to keep these structures in apposition. The parietal wound was closed without drainage. Strips of plaster were fixed firmly across the body from just below the nipples to the pelvic brim, in order to keep the parietes in contact with the liver and favour adhesions. The patient had hæmatemesis on the evening of operation, and died of shock and peritonitis three days afterwards.

*E*.—**Operations upon the Gall-bladder.**—CASES 22, 45, and 50.

I have so recently dealt with the whole subject of the surgery of the liver, gall-bladder, and biliary passages,[1] and with questions relating to the diagnosis of enlargements of the gall-bladder and the technique of cholecystotomy,[2] that I need not dwell upon these matters in this paper. There are, however, one or two points that are worth reflection. In the first place, it is not sufficiently widely recognised that the dangers of a **distended gall-bladder** are great enough to make it desirable to operate upon all cases of enlarged gall-bladder—unless obviously due to malignant disease—whether there be jaundice or not, and whether there be pain or not. In the next place, it is impossible in many cases of jaundice associated with enlarged gall-bladder to be certain of the diagnosis, and an exploratory operation should be undertaken under such circumstances much more frequently than has been done in the past. Gallstones commonly cause so much inflammatory thickening in the walls of the gall-bladder, and so many surrounding adhesions from local peritonitis, that jaundice produced by cholelithiasis is frequently associated with a contracted gall-bladder. On the other hand, jaundice produced by malignant disease leads to a rapid distension of the gall-bladder with bile before adhesions have time

[1] *Practitioner*, 1900, lxv. 535. [2] *Brit. M. J.*, 1900, i. 1329.

to form, and is therefore usually associated with an enlarged gall-bladder. There are, however, many exceptions to this general statement, and hence the desirability of the exploratory operation which has been referred to. Case 22 is to the point. The patient was deeply and persistently jaundiced, the gall-bladder was greatly distended, and the patient had lost four stone four pounds in weight. Such a condition might easily have been mistaken for malignant disease, but by operation I removed nine and a-half ounces of bile-stained fluid and forty-six gallstones, and the patient made a good recovery.

Case 45 presented no unusual features. I performed cholecystotomy, and removed sixty-two fragments of calculi—one stone being three-quarters of an inch in diameter.

(*To be continued*).

## Figures and Tables

**Fig. 1. f1:**